# A CBL-interacting protein kinase *AdCIPK5* confers salt and osmotic stress tolerance in transgenic tobacco

**DOI:** 10.1038/s41598-019-57383-x

**Published:** 2020-01-15

**Authors:** Naveen Kumar Singh, Pawan Shukla, P. B. Kirti

**Affiliations:** 1 Department of Plant Sciences, School of Life Sciences, University of Hyderabad, Hyderabad, 500046 India; 2 Agricultural Research Organization-the Volcani Center, 68 HaMaccabim Road P.O.B 15159, Rishon LeZion, 7505101 Israel; 3 Central Sericultural Research and Training Institute, Central Silk Board, NH-1A, Gallandar, Pampore, 192121 J & K India; 4 Agri Biotech Foundation, Rajendranagar, Hyderabad, 500030 India

**Keywords:** Plant molecular biology, Plant stress responses

## Abstract

CBL interacting protein kinases play important roles in adaptation to stress conditions. In the present study, we isolated a CBL-interacting protein kinase homolog *(AdCIPK5)* from a wild peanut (*Arachis diogoi*) with similarity to *AtCIPK5* of Arabidopsis. Expression analyses in leaves of the wild peanut showed *AdCIPK5* induction by exogenous signaling molecules including salicylic acid, abscisic acid and ethylene or abiotic stress factors like salt, PEG and sorbitol. The recombinant AdCIPK5-GFP protein was found to be localized to the nucleus, plasma membrane and cytoplasm. We overexpressed *AdCIPK5* in tobacco plants and checked their level of tolerance to biotic and abiotic stresses. While wild type and transgenic plants displayed no significant differences to the treatment with the phytopathogen, *Phytophthora parasitica* pv *nicotianae*, the expression of *AdCIPK5* increased salt and osmotic tolerance in transgenic plants. Analysis of different physiological parameters revealed that the transgenic plants maintained higher chlorophyll content and catalase activity with lower levels of H_2_O_2_ and MDA content during the abiotic stress conditions. *AdCIPK5* overexpression also contributed to the maintenance of a higher the K^+^/Na^+^ ratio under salt stress. The enhanced tolerance of transgenic plants was associated with elevated expression of stress-related marker genes; *NtERD10C*, *NtERD10D*, *NtNCED1*, *NtSus1*, *NtCAT* and *NtSOS1*. Taken together, these results indicate that AdCIPK5 is a positive regulator of salt and osmotic stress tolerance.

## Introduction

Plants have developed an elaborate network of signaling pathways to counter the challenges posed by various stressful environmental conditions. In general, Ca^2+^ serves as an ubiquitous secondary messenger that is reported to be involved in several biological processes including abiotic stress responses, pathogen defense and ion homeostasis adjustment^1^. Studies on Ca^2+^ dynamics have indicated stimulus-specific elevations in cytosolic Ca^2+^ concentration, termed as ‘calcium signatures’. To decode each signature, cells retain precise tools and mechanisms that include Ca^2+^ sensors and their downstream target proteins^2^. The sensor proteins exhibit a Ca^2+^ binding site in their helix-loop-helix region^3^. These proteins are classified into two categories as sensor responders and sensor relays^4^. Sensor responders such as Ca^2+^ dependent protein kinases (CDPKs) contain both Ca^2+^ binding and kinase activities, while sensor relays like calmodulin (CaM) and calmodulin-like proteins (CML) do not exhibit kinase activity. However, after binding with Ca^2+^, they interact with other protein kinases to regulate their activities^5^.

Compared to other organisms, the Ca^2+^ signaling mechanism in plants is more complex with the acquisition of several specific Ca^2+^ sensor proteins. The calcineurin B-like protein (CBL) family is one of these specific sensor proteins. Arabidopsis CBL4/SOS3 is the first CBL protein reported from the plant system^6^. Since then, several CBL proteins have been identified from different plants^7^. These proteins act as sensor relays and target a family of Ser/Thr kinases known as CBL-interacting protein kinases (CIPKs). Like CBLs, CIPKs are unique to the plant system^8^. Genome-wide studies have identified 26 CIPKs in Arabidopsis, 31 in rice, 27 in poplar, and 32 in sorghum^7^. The overall structure of CIPKs includes a catalytic N-terminal domain and a regulatory C-terminal domain^1,9^. Within the regulatory domain, a highly conserved stretch of 24 amino acids has been designated as NAF domain. It is believed to be responsible for mediating the CIPK interaction with CBL proteins^10^.

Many CIPKs have been functionally characterized in the recent past. For example, Arabidopsis AtCIPK24/SOS2 was initially identified as an AtCBL4 target protein that participated in salt tolerance mechanism by activating the Na^+^/H^+^ antiporter located on the root plasma membrane^11,12^, while AtCBL10-AtCIPK24 system was shown to be involved in the maintenance of Na^+^ homeostasis in shoots and leaves^13^. Moreover, reports suggest that AtCIPK24 might also be involved in activating the H^+^/Ca^+^ antiporter to maintain the intracellular Ca^2+^ levels^14^. The role of AtCBL1-AtCIPK1 complex was identified in ABA-dependent stress responses, whereas AtCBL9-AtCIPK1 complex appeared to modulate ABA-independent stress responses^15^. AtCBL9-AtCIPK3 complex was shown to be a negative regulator of ABA responses during seed germination^16^. CIPKs were also thoroughly studied in other plant species such as maize, rice, soybean and sorghum^7^. The transcript level analysis of *ZmCIPK16* in maize seedlings showed its strong upregulation by Polyethylene glycol (PEG), ABA, NaCl, dehydration, heat, and drought^17^. The expression of *PsCIPK* gene in *Pisum sativum* was induced in various abiotic and biotic stresses but not by dehydration and ABA treatments^18^. A *CaCIPK*2*5* gene from chickpea was reported to enhance root growth along with dehydration and salt stress tolerance in transgenic tobacco plants^19^.

Peanut (*Arachis hypogaea*) is an important oil crop grown worldwide for its oil and protein. Its productivity is greatly influenced by various environmental stresses. However, studies have shown that its wild relatives exhibit the high level of tolerance to various biotic and abiotic stress conditions^20^ and *Arachis diogoi* is one of them. In comparison to others, very little is known about the CIPKs from *Arachis* species. In the present study, using the available partial cDNA sequence of AdDR-5 (NCBI Accession No. EF371923) identified in a differential gene expression study of *Arachis diogoi* treated with the fungal pathogen, *Phaeoisariopsis personata*^21^, a full length cDNA was cloned and named as *AdCIPK5*. Its transcript levels were analyzed in *Arachis diogoi* during various treatments. Tobacco transgenic plants overexpressing *AdCIPK5* were checked for tolerance against various stress treatments. These observations are reported in this communication.

## Results

### Isolation of full length cDNA and sequence analysis

A full length cDNA of 2,031 bp was isolated by using the RACE-PCR approach. Sequence analysis of this revealed the presence of 1,386 bp long ORF region along with 408 bp of 5′ and 237 bp of 3′UTRs in the cDNA (Fig. S1). The ORF encodes a 461 amino acid polypeptide. The encoded protein had a predicted molecular mass of ~52 kDa with the isoelectric point value of 8.75. The SignalP analysis did not show any signal peptide in the deduced amino acid sequence. The amino acid sequence exhibited 62% and 99% similarity with CIPK5 protein from *Arabidopsis thaliana* and *Arachis duranensis*, respectively. Hence, this cDNA was designated as *AdCIPK5*. A Blast analysis revealed the presence of conserved N-terminal activation loop and C-terminal NAF domain in the predicted protein sequence (Fig. S2). Like other CIPKs, the activation loop of AdCIPK5 harbors three conserved amino acids, Serine (S), Threonine (T) and Tyrosine (Y) as possible target sites for phosphorylation by other protein kinases. Phylogenetic analysis showed the presence of two subgroups as intron-rich and intron-less in the phylogenetic tree (Fig. S3). AdCIPK5 was present in intron-less subgroup along with AduCIPK5, GmCIPK25, AtCIPK5, AtCIPK25 and AtCIPK16 as the closest ones.

### *AdCIPK5* expression analysis

**Figure 1:**
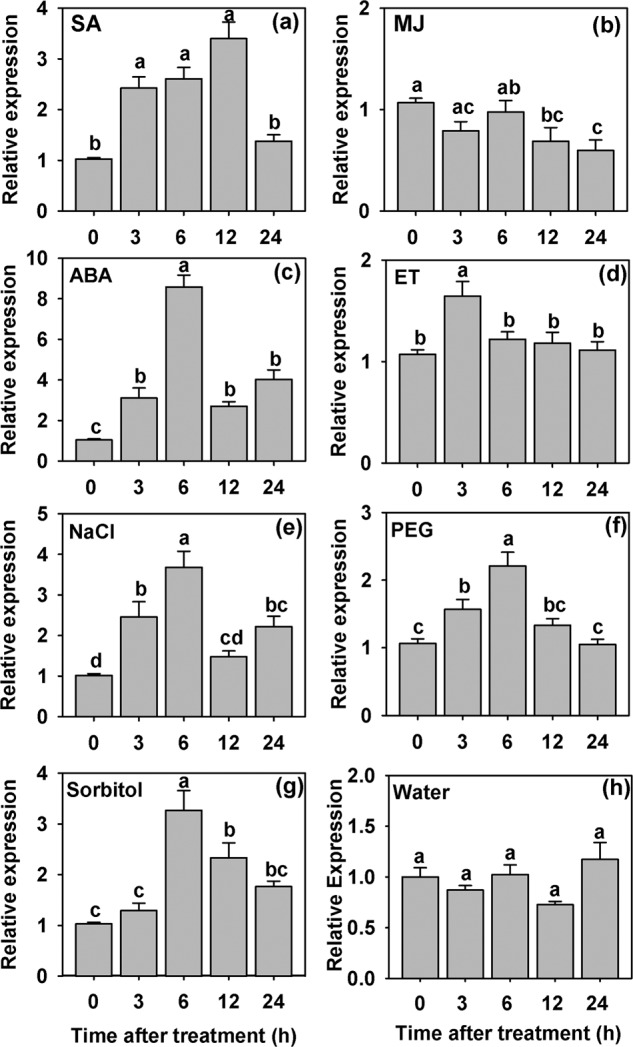
Expression analysis of *AdCIPK5* in mature leaf samples of *Arachis diogoi* under different treatments by qRT-PCR. (**a**) 500 μM Salicylic acid treatment; (**b**) 100 μM Methyl jasmonate treatment; (**c**) 100 μM, Abscisic acid; (**d**) 250 μM Ethephon treatment; (**e**) 200 mM NaCl treatment; (**f**) 10% Polyethylene glycol treatment; (**g**) 300 mM Sorbitol treatment; (**h**) water treatment. Error bar represents means ± SE (n = 3), and each replicate comprises 5 leaves. Different letters indicate significant difference (*P* ≤ 0.05) with each other.

The expression of *AdCIPK5* was analyzed in *A. diogoi* leaves in response to different phytohormones and stress treatments at various time point (Fig. 1). We used four basic phytohormones; Ssalicylic acid (SA), Methyl-jasmonate (MJ), Abscisic acid (ABA) and Ethephon (ET) in this study. These phytohormones are essential for plant growth and development and also mimic various stress conditions^22,23^. Our analyses showed that SA treatment caused early induction (at 3 h) of *AdCIPK5* but the level of expression (around 2-fold) was almost consistent between 3 to 12 h of treatment while it got downregulated later to the ground level at 24 h. It has been known that SA and MJ/ET associated molecular pathways work antagonistically to each other^24^. Therefore, we also checked the *AdCIPK5* expression during both MJ and ET treatments. MJ did not cause any induction whereas some upregulation was observed at 3 h of ET treatment. Interestingly, downregulation in *AdCIPK5* expression was observed at later stages of MJ treatment (12 and 24 hr). ABA is a major signaling molecule associated with abiotic stress responses. We observed that ABA treatment caused an early induction of *AdCIPK5* expression, which was nearly 8-fold higher at 6 h post-treatment. Even at the later time points (12 and 24 h), the expression remained around 2 to 3-fold higher to the basal level of expression. Compared to the marginal expression caused by other phytohormones, strong upregulation observed during ABA treatment suggests that ABA acts a major regulator of *AdCIPK5* expression.

Significant increase in *AdCIPK5* expression levels were also observed at different time points under NaCl, PEG and Sorbitol stress treatments. Under salt stress caused by NaCl, the expression level of this gene reached a maximum of around 3-fold higher compared to the initial level whereas PEG and Sorbitol enhanced its expression around 2-fold higher. Interestingly, the maximum expression levels were obtained at 6 h post-treatment, which was similar for all the treatments with the stressor molecules like the ABA treatment. The basal expression level of *AdCIPK5* was almost constant at different time points during the control (water) treatment (Fig. 1h).

### Subcellular distribution of AdCIPK5

**Figure 2:**
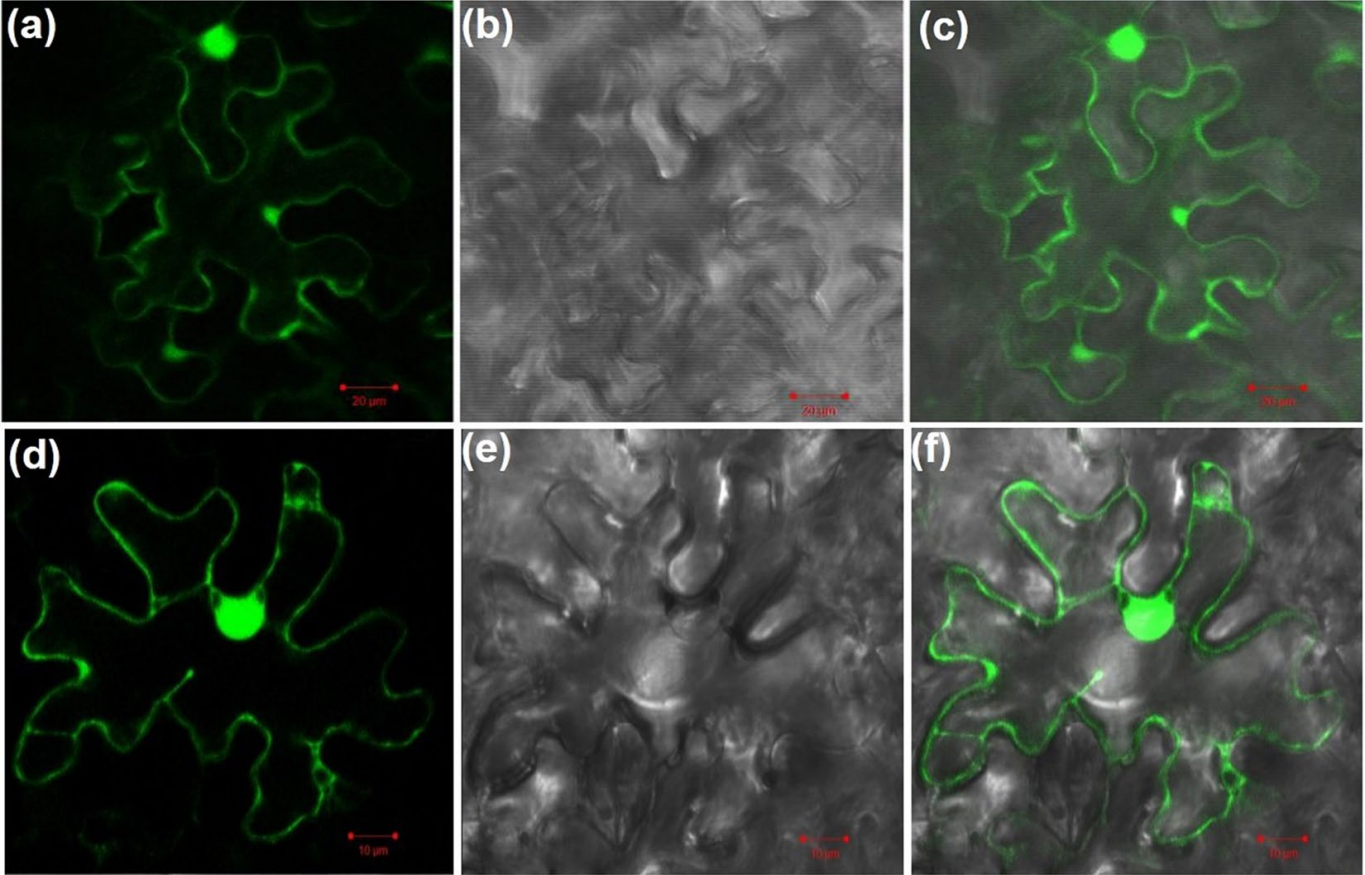
Subcellular localization of AdCIPK5. *N. benthamiana* leaves were infiltrated with Agrobacterium culture harboring empty vector pCAMBIA1302 and recombinant pCAMBIA1302-AdCIPK5 constructs for studying the subcellular localization of AdCIPK5. Leaf cells were visualized under a Confocal Laser Scanning Microscope. (**a**–**c**) Control pCAMBIA1302 vector showing GFP throughout the cell. (**d**–**f**) pCAMBIA1302-AdCIPK5 recombinant vector showing GFP expression in nucleus and plasma membrane with some presence in cytoplasm.

The subcellular localization of AdCIPK5 was studied by constructing a C-terminal translational fusion of green fluorescent protein (GFP) with AdCIPK5 and expressing the fusion gene transiently in *Nicotiana benthamiana* leaves through Agroinfiltration method. A control vector overexpressing GFP only (35S:GFP) was used as a control. After 48 h incubation at room temperature, the localization of the fusion protein was observed under confocal microscopy. When GFP alone was transiently expressed, the GFP fluorescence was visualized throughout the cell, while the recombinant AdCIPK5:GFP protein was appeared to be localized mainly in nucleus and on the plasma membrane with some fluorescence being observed in cytoplasm as well (Fig. 2).

### PCR and RT-PCR analysis of putative transgenic plants

Nine independent primary transgenic tobacco plants showed the expected amplification of 700 bp of *nptII* and 1383 bp of *AdCIPK5*, respectively in PCR analysis (Fig. S4). In a semi-quantitative RT-PCR, the putative transgenic plants 1 and 6 showed highest expression level of *AdCIPK5* while the transgenic plant 2 was with lowest expression level (Fig. S5a). *Actin* amplification was used as the internal control. The high expression plants (1 and 6) exhibited the expected 3:1 segregation for resistance to the selection antibiotic (Kanamycin) in T_1_ generation. Homozygosity of the transgenic lines was checked by 100% seed germination on kanamycin selection medium in T_2_ generation. Based on these results, line 1 and 6 (high expression) and line 2 (low expression) plants were used for subsequent analyses. The transcript level *AdCIPK5* in these three lines and wild type (WT) was also determined in T_2_ generation (Fig. S5b). In the present work, line 1, 6 and 2 were represented as H1, H2 and Low respectively.

### Biotic stress analysis

WT and transgenic T_2_ plants were used in detached leaf treatments with the plant pathogen *Phytophthora parasitica* pv *nicotianae*. This pathogen causes severe damage to tobacco plants and has been used in plant resistance analysis in various studies^25,26^. We observed the infection symptoms after 2 days post inoculation (dpi) and after 5 dpi, and necrotic areas covered almost complete surface of WT and transgenic leaves (Fig. S6a). The level of resistance was measured by calculating the percentage diseased leaf area (DLA) and by measuring the cell death in leaves caused due to pathogen treatment. Our analysis revealed that around 90% of all the tested leaves were damaged after 5 dpi (Fig. S6b). Further, the Evans blue staining of infected leaves also showed similar cell death in WT and transgenic lines (Fig. S6c) suggesting that the level of *AdCIPK5* expression in transgenic tobacco had no significant effect on imparting resistance to the test pathogen in tobacco.

### Seed germination analysis

**Figure 3:**
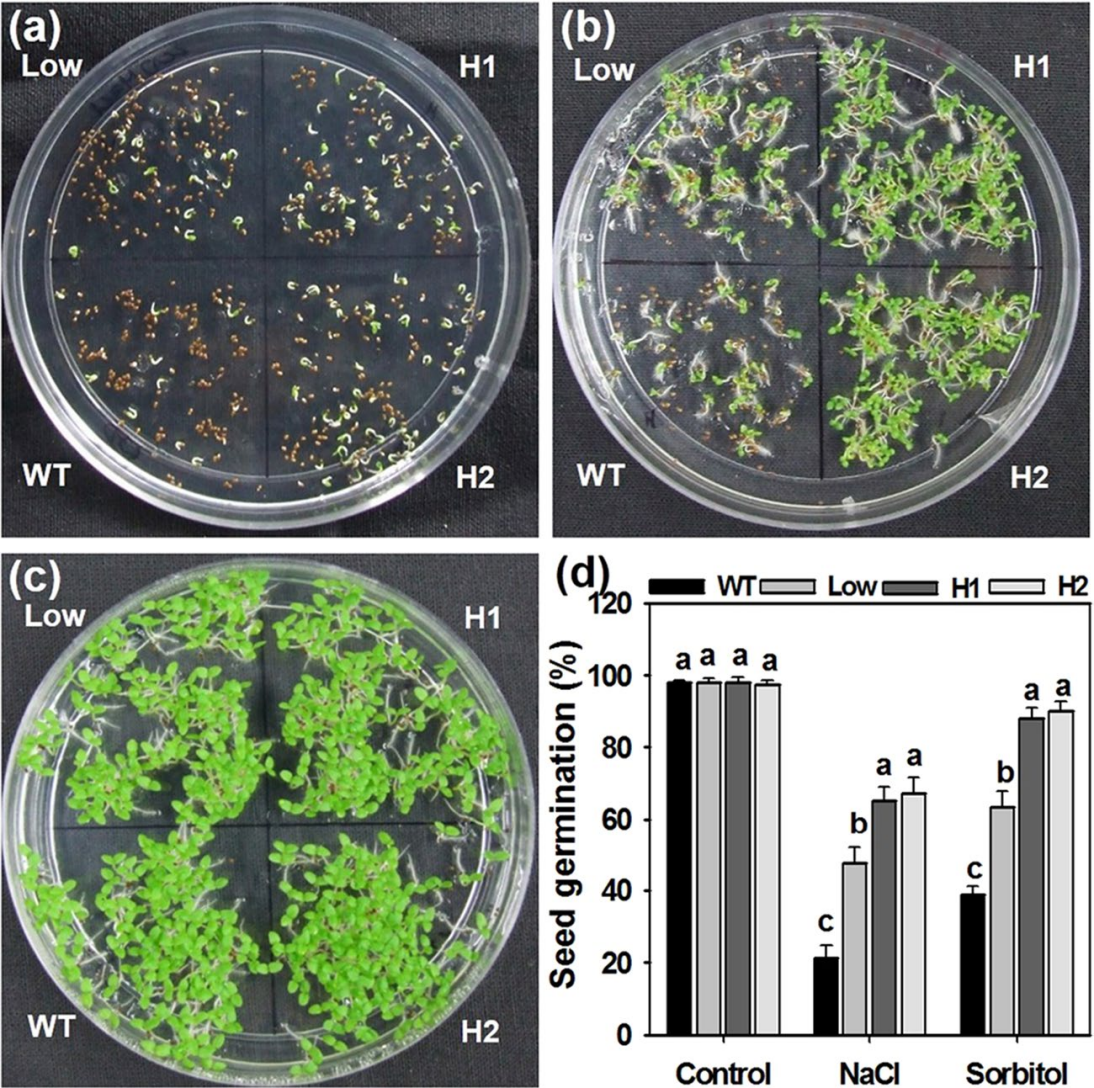
Seed germination assay under salt and osmotic stress. The germination rate of WT and transgenic seeds was detected on a medium supplemented with (**a**) 200 mM NaCl and (**b**) 300 mM Sorbitol for 7 d. (**c**) Control plate without any stress treatment was maintained for the same period of time. (**d**) Graphical representation of percentage seed germination after 7 d NaCl and osmotic stress treatments. H1 and H2 represent high expression lines whereas Low represents low expression line seedlings. Values are means ± SE (n = 3), and each replicate comprises 150 seeds. Different letters indicate significant difference (*P* ≤ 0.05) with each other.

Seeds of the WT and transgenic plants were surface sterilized and plated on half strength MS medium containing 200 mM NaCl and 300 mM Sorbitol (Fig. 3). Medium lacking the stressor agents served as the control. After germination for 7 d, the seeds that developed green cotyledons were scored on the stress media. Out of 150 seeds taken for analysis, almost 98 to 100% seeds germinated on the control plates from all the lines. However, the germination percentage on the medium containing 200 mM NaCl was around 20% for WT while the low expression line exhibited 50% and it was 70% for both the high expression lines H1 and H2. Similarly, 40–45% of WT, 70% of low and almost 90–95% seeds of H1 and H2 germinated on 300 mM Sorbitol containing medium after 7 d treatment. These results demonstrated a higher level of tolerance in transgenic lines against abiotic stress conditions like salt and osmotic stress during the seed germination stage.

### Salt stress tolerance at the seedling stage

**Figure 4:**
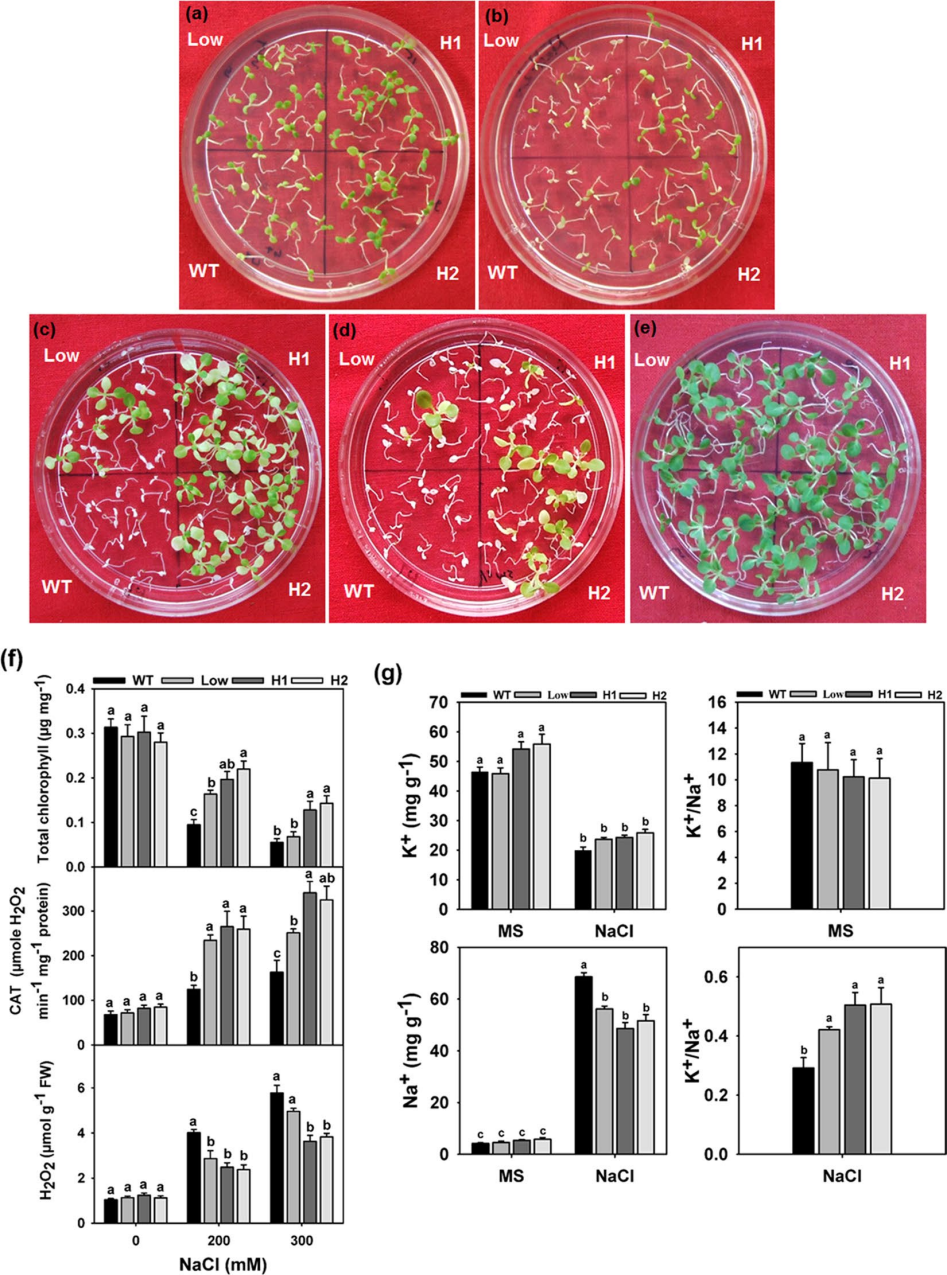
Effect of NaCl stress on seedlings growth. The 10 d old seedlings were treated with NaCl. Clear differences were observed between WT and transgenic seedlings after 10 d of 200 mM (**a**) and 7 d of 300 mM (**b**) NaCl treatment. Seedlings on NaCl-free medium; recovery (for 15 d) after 6 d treatment with 200 mM and 300 mM NaCl (**c,d**). (**e**) Unstressed seedlings were regularly sub-cultured along with the NaCl-treated seedlings. (**f**) Total chlorophyll content, CAT activity and H_2_O_2_ generation was measured after 6 d treatment. Values are means ± SE (n = 3) and each replicate comprises a minimum of 10 seedlings. (**g**) Measurement of K^+^ and Na^+^ content under NaCl treatment after 6 d. The whole seedlings of the WT and transgenic lines were sampled to detect K^+^ and Na^+^ contents, and then the ratio of K^+^ and Na^+^ was calculated. Values are means ± SE (n = 3), and each replicate comprises minimum 20 seedlings. Different letters indicate significant difference (*P* ≤ 0.05) with each other. H1 and H2 represent high expression lines whereas Low represents low expression line seedlings.

The 10 d old seedlings of the WT and transgenic lines germinated on stress free medium were transferred to different concentrations (200 and 300 mM) of NaCl. After 10 d treatment, WT seedlings from 200 mM NaCl medium displayed higher chlorosis and retarded root elongation compared to the transgenic lines. Chlorosis was also observed in low expression line seedlings, whereas both H1 and H2 developed normally without significant chlorosis (Fig. 4a). After 7 d treatment with 300 mM NaCl, the WT and low expression line seedlings were severely affected (Fig. 4b). However, the high expression lines showed better growth with mild chlorosis. The ability of treated seedlings to recover from stress condition was also checked by transferring them from salt to salt free medium. Considering the severity of damage to the seedlings due to the stress condition, we chose to transfer the seedling after 6 d of treatment from both 200 mM and 300 mM NaCl media to the recovery plates and then observed their recovery for 15 d (Fig. 4c,d). Control seedlings were also maintained simultaneously (Fig. 4e). We observed complete bleaching of WT seedlings on both the recovery plates, whereas the high expression lines from 200 mM media were able to recover with true leaf and root formation while the 300 mM concentration of NaCl appeared to be too high even for the high expression transgenic lines, which was evident from only 30 to 40 percent recovery of H1 and H2 lines.

Further, the estimation of various physiological parameters revealed that the transgenic seedlings retained higher chlorophyll content and catalase (CAT) activity with lower H_2_O_2_ generation compared to WT on 200 mM NaCl containing medium. On 300 mM medium, only the high expression lines retained significantly higher chlorophyll content and CAT activity with lower H_2_O_2_ generation (Fig. 4f). In addition, the effect of *AdCIPK5* overexpression on Na^+^ and K^+^ accumulation in the transgenic plants was also estimated during 200 mM salt treatment (Fig. 4g). In the absence of any stress, all the samples showed almost similar Na^+^ and K^+^ levels whereas transgenic seedlings from NaCl treatment showed significantly lower Na^+^ levels compared to WT with no apparent change in the K^+^ levels. Hence, the transgenic seedlings were able to maintain higher K^+^/ Na^+^ ratio on NaCl treatment compared to the control.

### Osmotic stress tolerance

**Figure 5:**
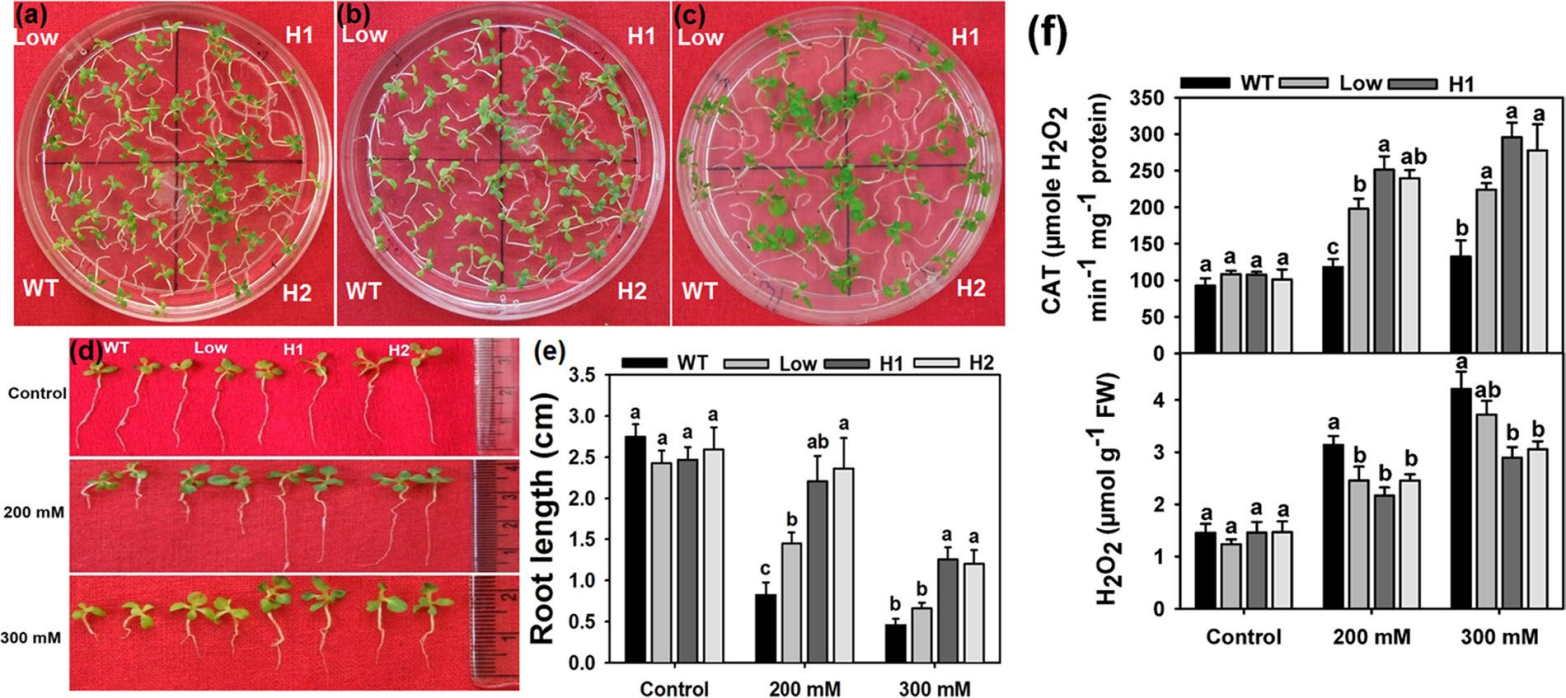
Effect of osmotic stress on seedling growth. The 10 d old WT and transgenic seedlings were transferred to 200 mM (**a**) and 300 mM (**b**) Sorbitol medium for 15 days. (**c**) Seedlings not subjected to stress. (**d**) Phenotypic difference in the growth of seedlings after 15 d of treatment. (**e**) Graphical representation of root length measurements. Values are means ± SE (n = 3), and each replicate comprises 15 seedlings. (**f**) Measurement of CAT activity and H_2_O_2_ content after 10 d of stress treatment. Values are means ± SE (n = 3), and each replicate comprises minimum 10 seedlings. Different letters indicate significant difference (*P* ≤ 0.05) between each other. H1 and H2 represent high expression lines whereas Low represents low expression line seedlings.

To check the osmotic stress response of the transgenics at seedling stage, the 10 d old WT and transgenic seedlings were transferred to media containing 200 and 300 mM Sorbitol and observations were made after 15 d of treatments with appropriate control (Fig. 5a–c). We observed that the growth of transgenic lines on both 200- and 300-mM sorbitol plates was near normal and better than the WT seedlings. The differences in growth patterns were clearly depicted in Fig. 5d. Further, the measurement and graphical representation of root length of the treated seedlings of all the lines clearly showed that transgenic lines performed better with higher root length compared to the WT under two levels of osmotic stress (Fig. 5e). However, on 300 mM sorbitol, the root length of the low expression line seedlings was also suppressed similar to the WT. Further on, analysis of CAT activity and H_2_O_2_ generation revealed that the transgenic seedlings exhibited higher CAT activity with lower H_2_O_2_ generation on stress media (Fig. 5f).

### Leaf Disc assay for salt and osmotic stress

**Figure 6:**
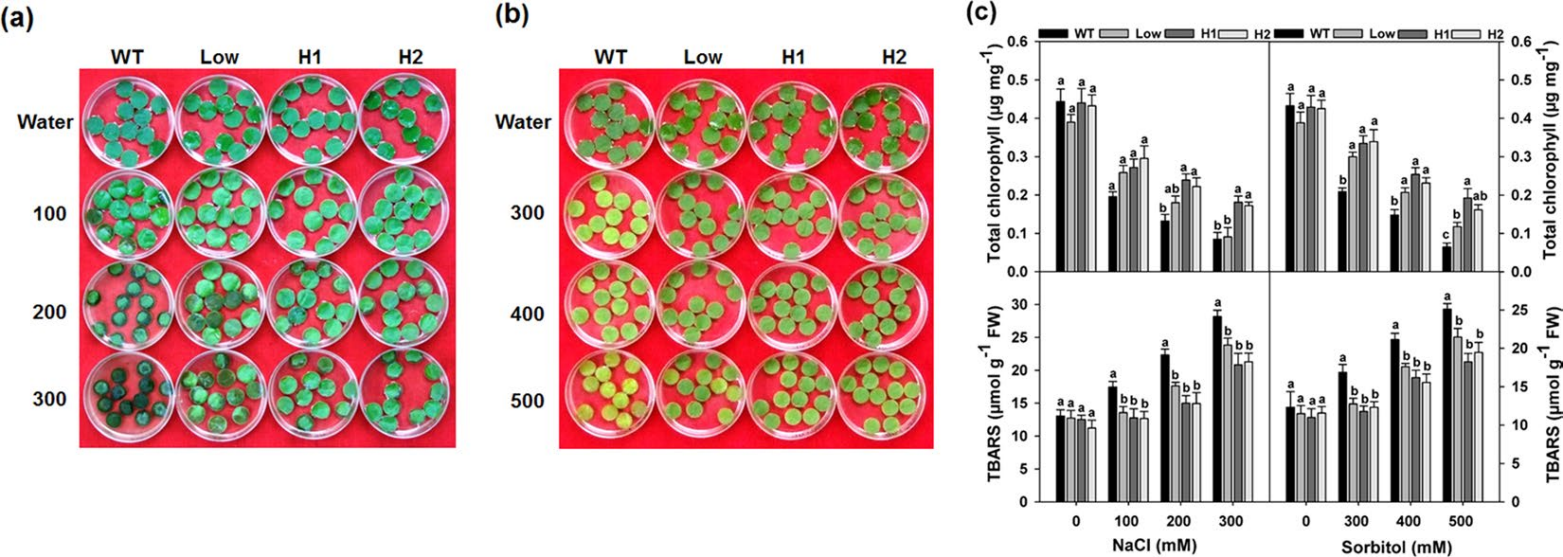
Leaf disc assay. (**a**) Stress tolerance exhibited by the leaf discs of WT and transgenic lines at various concentrations of NaCl (100, 200 and 300 mM) and (**b**) sorbitol 300, 400 and 500 mM treatments. Photographs were taken post 3 d NaCl and 5 d Sorbitol treatments. (**c**) Total chlorophyll and TBARS were measured in WT and transgenic leaf discs after 2 d NaCl treatment and after 4 d Sorbitol treatment. Values are means ± SE (n = 3), and each replicate comprises minimum 15 leaf discs. Different letters indicate significant difference (*P* ≤ 0.05) between each other. H1 and H2 represent high expression lines whereas Low represents low expression line seedlings.

To assess the stress tolerance at mature leaf stage, a leaf disc assay was performed using different concentrations of NaCl and Sorbitol. Leaf discs from 7-week-old plants were used in both the treatments. NaCl tolerance in the discs of transgenic plants was observed after 3 d treatment (Fig. 6a). Chlorophyll estimation revealed a dose dependent loss of total chlorophylls in the leaf discs of the WT plants compared with those from the transgenic plants, which were able to retain significantly higher total chlorophyll content (Fig. 6c). However, a non-significant difference was observed between WT and low line plants on the 300 mM medium. Similarly, the levels of Thiobarbituric acid reactive substances (TBARS) increased in the WT compared to the transgenic lines. In case of Sorbitol treatment, chlorosis started appearing after 3 d treatment (data not shown), which became more prominent after 5 d treatment in WT plants with increasing concentration of Sorbitol, whereas the leaf discs of transgenic plants showed reduced chlorosis (Fig. 6b). Leaf discs from distilled water treatment remained green in both WT and transgenic plants. Further, the chlorophyll content and lipid peroxidation levels (as evidenced by TBARS) of the leaf discs after 4 d treatment confirmed the observed phenotypic differences (Fig. 6c).

### ROS analysis

**Figure 7:**
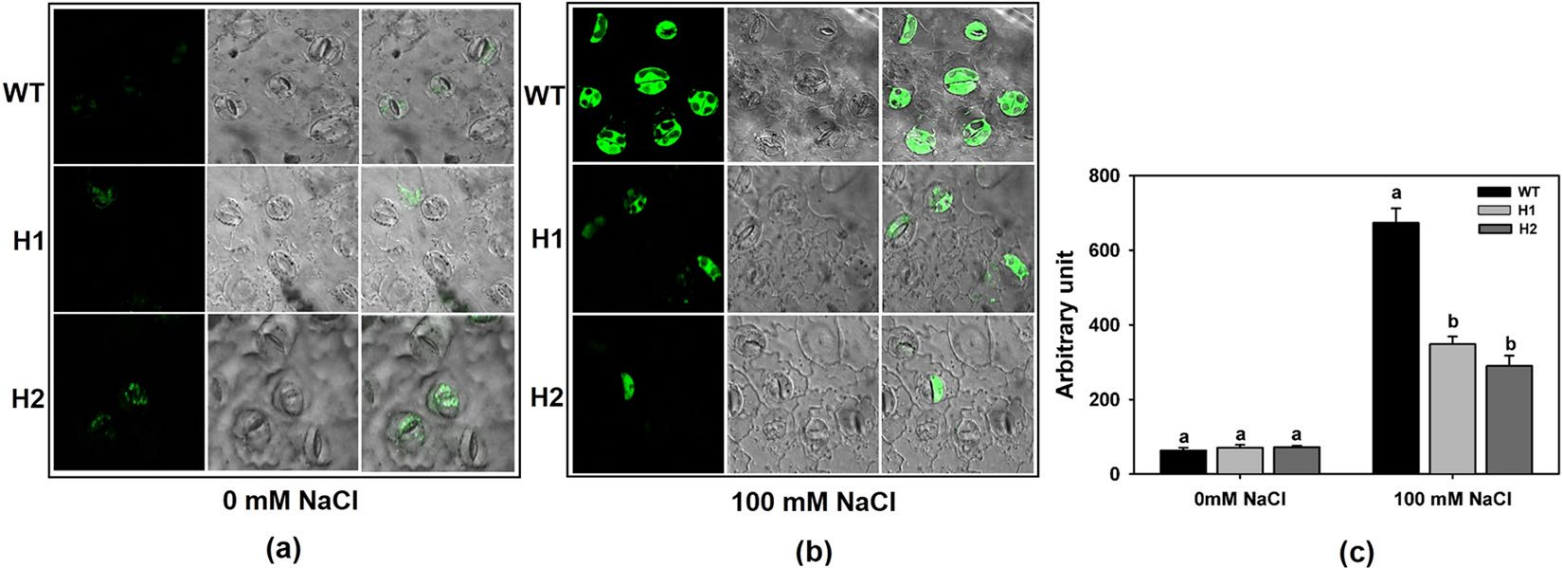
ROS detection in leaf epidermal guard cells using Confocal Microscopy. Fluorescence levels in WT and high expression line plants (H1 and H2) were observed after (**a**) control and (**b**) 100 mM NaCl treatments by staining with H_2_DCFDA. WT samples showed higher ROS accumulation during NaCl stress. Bright field images were also displayed. (**c**) Quantification of ROS production by using Image-J software. Values are means ± SE (n = 3), and each replicate comprises 100 stomata. Different letters indicate significant difference (*P* ≤ 0.05) between each other.

Both the high expression lines H1 and H2 along with the WT were used in the analysis of ROS generation (Fig. 7). In untreated control samples (without NaCl), the observed fluorescence due to the formation of Reactive Oxygen Species (ROS) as evidenced by the staining with 2ʹ,7ʹ-Dihydrodichlorofluorescein diacetate (H2DCFDA) in stomatal guard cells was minimal and more or less similar in WT and transgenic tobacco plants. Increased fluorescence as indicative of enhanced H_2_O_2_ formation was observed in guard cells of WT and transgenic plants in NaCl (100 mM) treatment. However, the fluorescence displayed by the transgenic plants was significantly less compared to the WT plants, which implied that there was reduced ROS formation in transgenic guard cells under sodium chloride stress.

### Transcript level analysis of stress related genes

**Figure 8:**
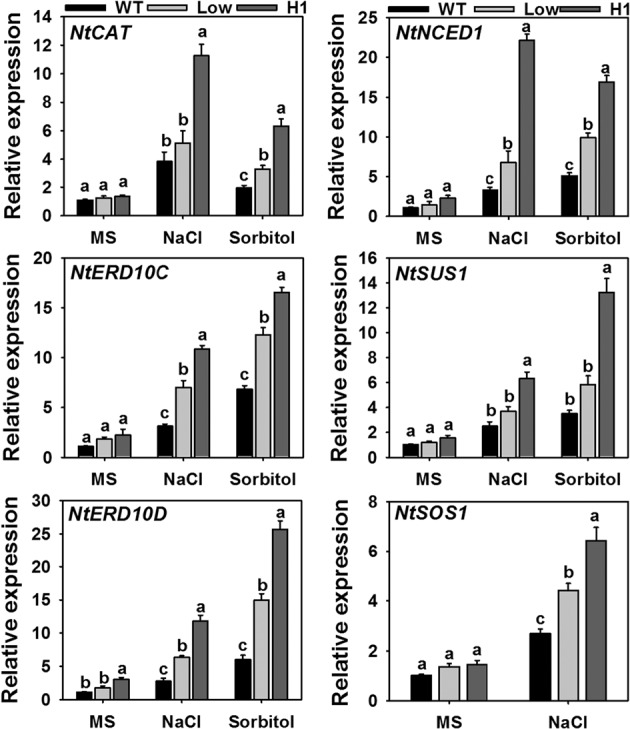
Transcript level analysis of stress-related genes in plants under NaCl and osmotic stress conditions. The 10 d old seedlings of WT and transgenic lines (low and H1) were exposed to NaCl treatment (200 mM NaCl) and osmotic stress treatment (300 mM Sorbitol) for 3 d followed by real time expression analysis of stress related genes. Values are means ± SE (n = 3), and each replicate comprises 5 seedlings. Different letters indicate significant difference (*P* ≤ 0.05) between each other.

To understand further the relative effect of *AdCIPK5* overexpression in salt and osmotic stress tolerance, the expression of different stress-related genes was analyzed in WT and the transgenic lines (Low and H1) with and without stress treatment (Fig. 8). The transcript level of six genes (*NtCAT, NtERD10C, NtERD10D, NtNCED1, NtSus1* and *NtSOS1*), which were reported to be involved in response to abiotic stress were analyzed. The 10 d old WT and transgenic seedlings grown in Petri dishes were exposed to salt (200 mM NaCl) and Sorbitol (300 mM) treatments for 3 d followed by a real time analysis of the expression of the above mentioned genes. The results showed that all stress-responsive genes analyzed were significantly induced in the high expression line compared to the control plants when exposed to salt and sorbitol treatments suggesting that *AdCIPK5* overexpression in tobacco has been associated with the upregulated expression of stress-related genes under salt and osmotic stress conditions.

## Discussion

In the present investigation, a full-length cDNA of *AdCIPK5* gene that was identified in differential gene expression analysis was cloned using the sequence of a partial cDNA from a wild peanut, *Arachis diogoi*^21^ using RACE-PCR approach. The analysis of deduced AdCIPK5 protein sequence showed the presence of highly conserved motifs such as the activation loop and NAF domain, which are typical of the CIPK family and are important for their plant function^1,10^. Phylogenetic analysis revealed the close homology of AdCIPK5 with AduCIPK5 of *Arachis duranensis*, GmCIPK5 of *Glycine max* and AtCIPK5, AtCIPK25, and AtCIPK16 of *Arabidopsis thaliana*. The Arabidopsis CIPKs exhibited roles in the plant developmental processes, salt stress responses and fungal infection^27–29^.

In the present study, we checked the quantitative expression of *AdCIPK5* in treatment with different phytohormones and stress inducers. The analysis showed that, the transcript levels were upregulated differentially at least at some point during the SA, ABA and ET treatments in *A. diogoi* leaves except MJ treatment. These hormones are regarded as essential components of plant adaptation system, but their responses are governed by complex interlinked mechanisms^22,23^. For example, during the pathogen attack, SA is associated with strong immune responses while JA/ET pathways act antagonistically to SA^24,30^. Similarly, ABA has long been known for its role in abiotic stress, but recent studies suggest that it can also regulate plant immunity in association with other hormones^31^. Since these phytohormones regulate the different sets of mechanisms, *AdCIPK5* induction can be assumed to be associated with multiple signaling pathways in the plant. Previous findings on CIPKs have confirmed their potential role in the abiotic stress tolerance^7^. Therefore, under stress conditions such as NaCl, Sorbitol and PEG, we examined the expression profile of *AdCIPK5*. During the treatments, the enhanced expression of *AdCIPK5* was observed in all the stress treatments, which suggested that the gene is positively involved in abiotic stress tolerance mechanisms.

CIPKs could be located at different sites within the cell^9^. For example, wheat proteins TaCIPK14 and TaCIPK29 have been observed across cells^32,33^. AtCIPK1 from Arabidopsis was found to be localized to the plasma membrane and to some extent also to the nucleus and cytosol, which was further recruited to the plasma membrane after interacting with AtCBL1 and AtCBL9^15^. Similarly, the AtCIPK5 was shown to be dynamically translocated from cytoplasm to tonoplast^34^. The analysis of the amino acid sequence revealed that the AdCIPK5 had no recognizable localization signal that was consistent with previous CIPKs findings^1^. However, with some presence in cytoplasm, the AdCIPK5:GFP fusion protein appeared to be located mainly in the nucleus and plasma membrane. Hence, it could be speculated that the role of AdCIPK5 is not limited to a specific organelle and that localization may depend on specific interacting partners.

As an active participant of Ca^+^2 signaling pathways, CIPKs play crucial roles in plant responses during the microbe and other pathogen attacks. An earlier study on tomato CIPK6/CBL10 complex reported its involvement in immune responses by generating ROS and ultimately leading to the programmed cell death in the plant^35^. Similarly, a TaCBL4/TaCIPK5 complex is involved in modulation of fungal resistance in wheat^36^. As elaborated in introduction, the partial cDNA sequence of *AdCIPK5* was initially identified during the fungal infection^21^. Further, in this study, the observed induction of *AdCIPK5* expression in response to SA, ABA, and ET suggested that this gene could be involved in plant defense mechanisms. Therefore, the transgenic tobacco plants overexpressing *AdCIPK5* were raised and their resistance was analyzed along with the WT in treatment with an oomycetes pathogen*, P. nicotianae*. Detached leaves were inoculated with the pathogen. The level of resistance in WT and transgenic leaves was determined by calculating the percentage diseased leaf area and by measuring the cell death caused by the *P. nicotianae* infection. No significant differences were observed in our analyses between WT and transgenic leaves. These findings suggested that the expression of AdCIPK5 did not enhance the pathogen resistance in tobacco transgenic plants. It is possible that the AdCIPK5 role is limited in pathogen resistance mechanisms with some unknown function. Likewise, rice OsCIPK14/15 also displayed a wide range of defence responses in cultured cells, but the adult plant showed no resistance to blast fungus^37^.

The functions of CIPKs in abiotic stress conditions have been widely studied. In Arabidopsis several CIPKs have been shown for their roles in regulating the stress responses^2,5^. In rice, overexpression of *OsCIPK3*, *OsCIPK12* and *OsCIPK15* significantly improved cold, drought and salt tolerance respectively^38^. Transgenic tobacco plants overexpressing *TaCIPK14* and *TaCIPK29* from wheat also conferred single or multiple stress tolerance^32,33^.

In the present investigation, we observed that the *AdCIPK5* transcript levels were upregulated during the abiotic stress treatments in the wild peanut. Therefore, using NaCl and Sorbitol, the WT and transgenic plants were tested for salt and osmotic stresses. The initial analysis was carried out at the stage of seed germination, which is considered to be the most crucial phase for the establishment of the plant and its final yield. Significantly, higher percentages of transgenic seed germination were recorded on both 200 mM of NaCl and 300 mM of Sorbitol media compared to WT indicating enhanced tolerance to both salt and osmotic stresses in tobacco transgenic plants expressing *AdCIPK5*. These transgenic tobacco lines were also able to perform better on both 200 and 300 mM NaCl containing medium at the seedling stage compared to the WT plants. However, the concentration of 300 mM of NaCl seemed to be not tolerable even for the transgenic plants. It was also confirmed on a medium of recovery where only 30–40 percent of high-expression seedlings could recover from NaCl stress of 300 mM, whereas most of them could recover from 200 mM level of stress. Similarly, during osmotic stress treatment, transgenic lines exhibited better growth patterns compared to their non-transformed WT counterparts. The transgenic seedlings showed higher root length than the WT on both 200 and 300 mM of Sorbitol stress media. However, a non-significant difference was observed between seedlings of WT and low-expression line on 300 mM medium.

Various physiological parameters were recorded to confirm the enhanced levels of abiotic stress tolerance exhibited by the transgenic plants ectopically expressing *AdCIPK5*. Both NaCl and osmotic stresses affect nearly all plant growth and developmental stages resulting in the generation of excessive reactive oxygen species (ROS) that can cause damage by oxidizing various vital cell components such as proteins, lipids and DNA^39^. To survive under the NaCl stress, plants must sequester to an appropriate cellular compartment or expel the excessive Na^+^ and therefore, maintain a higher K^+^ level, which ultimately helps in scavenging higher ROS levels^40,41^. In comparison to the WT under NaCl stress, the *AdCIPK5* transgenic seedlings showed better ability to retain higher K^+^/Na^+^ ratio. This observation was supported by other parameters like higher chlorophyll content, higher CAT activity with reduced levels of H_2_O_2_ in the transgenic plants. These parameters are important in assessing the ability of the plant to sustain itself against the impending stresses using the antioxidant defence mechanisms^42^. Similarly, with increased CAT activity and lower H_2_O_2_ content, the *AdCIPK5* transgenic seedlings maintained better growth during the osmotic stress conditions also. Furthermore, the WT and transgenic plants were also evaluated against stress conditions at a mature stage. For this purpose, leaf discs from seven week-old pot grown plants were treated with different NaCl and Sorbitol concentrations. Total chlorophyll content and TBARS values were analyzed in all the treated samples. Analysis of TBARS is used to assess the ROS-mediated membrane damage^43^. Transgenic lines were able to retain higher chlorophyll content compared to WT plants during both the treatments with significantly reduced TBARS levels. This clearly indicated reduced lipid peroxidation and membrane damage in *AdCIPK5* overexpression lines that encountered stress treatments. Direct ROS level measurement is used as an effective index to evaluate multiple stress tolerance in plants^42^. For example, higher ROS levels were recorded under salt stress in Arabidopsis *Atcipk24* mutant while *ZmCIPK21* overexpressing lines in maize exhibited lower levels of ROS compared to the control plants^44,45^. In our study, staining with a ROS sensitive fluorescent dye, H_2_DCFDA was used during NaCl treatment to monitor the visible differences in ROS levels in stomatal guard cells. The results showed that there was significantly reduced fluorescence in tobacco transgenic plants, which could be associated with the constitutive expression of *AdCIPK5*. No major differences in control samples were observed. Taken together, these observations clearly indicate that AdCIPK5 functions in a pathway that controls ROS detoxification in response to NaCl and osmotic stresses.

To understand the function of AdCIPK5 in regulation of gene expression under NaCl and osmotic stresses, the transcript levels of several stress-related marker genes were evaluated in transgenic plants expressing *AdCIPK5* ectopically. Results clearly showed that the expression of genes *NtERD10C*, *NtERD10D*, *NtNCED1*, *NtSus1*, *NtCAT* and *NtSOS1* was significantly higher in the high expression line plants compared with the WT under NaCl stress. These genes have been reported earlier to contribute to mechanisms of plant stress tolerance. Both *NtERD10C* and *NtERD10D* encode LEA proteins that act as macromolecule stabilizers and membrane protectants while *NtNECD1* is an important gene of the ABA biosynthesis pathway^46,47^. In stress conditions, *SUS1* plays a crucial role in osmotic maintenance^48^. *CAT* is a catalase that assists the cell in scavenging excessive H_2_O_2_^49^. *SOS1* encodes a putative Na^+^/H^+^ antiporter that modulates the ion movement across the membrane and is involved in the mechanism of salt tolerance^12^. Similarly, the higher expressions of *NtERD10C*, *NtERD10D*, *NtNCED1*, *NtSus1* and *NtCAT* were also observed during osmotic stress conditions. These findings suggest that by modulating the expression of stress-related genes, AdCIPK5 maintains the cellular homeostasis in NaCl and osmotic stress conditions. Further studies are required for further insights into the mechanism by which AdCIPK5 regulates the associated genes with stress responses.

In conclusion, *AdCIPK5* is clearly identified as a stress-responsive gene by our results. The gene was reported during the fungal infection but our analysis showed its involvement in abiotic stress tolerance. This can be explained on the basis of previous findings suggesting that CIPKs are not very specific to their role. By forming different sets of CBL-CIPK complex, they can participate in different stress tolerance mechanisms^5,50^. Despite extensive studies of the CBL/CIPK network in Arabidopsis and other plants, their identities and roles in *Arachis diogoi* are largely unknown. Therefore, the identification of AdCIPK5 target proteins will provide more insights in understanding its regulatory mechanism in response to NaCl and osmotic stresses. The high similarities between AdCIPK5 and the CIPK5 of *Arachis duranensis* whose genome has been sequenced recently^51^ would help unravel the AdCIPK5 interacting partners in peanuts in future investigations. Taken together, *AdCIPK5* overexpression conferred tolerance to NaCl and osmotic stresses at various stages of growth in transgenic tobacco. To our best knowledge, this is the first report of the functional characterization of any CIPK gene from *Arachis* spp. We believe that our results would help in understanding the role that CIPK family proteins play in plant stress management.

## Materials and Methods

### Plant materials and treatments

Wild peanut (*Arachis diogoi*, ICG8962) and tobacco (*Nicotiana tabacum* var Samsun) plants were maintained in the green house. Detached leaves of *A. diogoi* were utilized for different treatments, and the experiments were performed essentially as described earlier^52^. The leaves were used in various treatments, 500 μM salicylic acid (SA), 100 μM methyl jasmonate (MeJA), 100 μM abscisic acid (ABA), 250 μM ethephon, 200 mM NaCl, 300 mM Sorbitol and 10% PEG, while the treatment with water served as a control. Samples were collected at regular intervals, quick-frozen in liquid nitrogen, and stored at −80 °C.

### RACE and isolation of full length cDNA

Rapid amplification of cDNA ends (RACE) reaction was performed to obtain full length cDNA of *AdCIPK5* from the partial sequence identified earlier^21^ by using SMARTer™ rapid amplification of cDNA ends (RACE) Kit (Clontech, USA) following the manufacturer’s instructions. All the reactions were performed using hot-start DNA polymerase provided along with the kit. The gene specific primers (GSPs) used for 5′/3′ RACE-PCR reaction were provided in Table S1. The full-length cDNA sequence of *AdCIPK5* was obtained by aligning 5′/3′ RACE products and the partial AdDR-13 cDNA sequences. The open reading frame (ORF) of the *AdCIPK5* sequence was amplified with ORF-F1 and ORF-R1 primers (Table S1) by using Phusion™ high fidelity DNA polymerase (Finnzymes, NEB, UK). The amplified PCR products were cloned in pTZ57R/T vector and sequenced commercially for the confirmation.

### Analysis of cDNA and protein sequence

Basic sequences were analyzed and compared by using BLASTn and BLASTp similarity searches. ExPASy tools were used for nucleotide translations, isoelectric point prediction and molecular mass calculations. Multiple sequence alignment and phylogenetic tree construction were performed using ClustalW and MEGA 6 software respectively. Signal peptide prediction was done using SignalP 4.1.

### Real time gene expression analysis

The qRT-PCR reaction was performed essentially as described earlier^50^. Alcohol dehydrogenase class III (*adh3*) and Ubiqutin (*Ubq*) genes were used as internal controls for *Arachis diogoi* and tobacco samples respectively^53,54^. The ΔΔC_T_ method was used for the estimation of relative fold change in RNA expression. Primers used in this study were provided in Table S2.

### AdCIPK5 subcellular localization analysis

The *AdCIPK5* ORF was reamplified by using ORF-F1 and ORF-R1 primers with *Nco*I and *Spe*I restriction sites respectively. The amplified product was digested and cloned in binary vector pCAMBIA 1302, digested with the same set of enzymes. Further, the confirmed recombinant vector was mobilized into *Agrobacterium* strain, *EHA*105 using the standard freeze thaw method. Similarly, the empty 1302 vector was also mobilized into the same *Agrobacterium* strain. *Nicotiana benthamiana* leaves were used for agroinfiltration^21^. The GFP expression was visualized in infiltrated leaves using laser scanning confocal microscopy (Leica TCS SP2 with Leica DM6000 microscope).

### Construct preparation and tobacco transformation

The *AdCIPK5* ORF was reamplified with ORF-F2 and ORF-R2 primers harboring *Apa*I and *Kpn*I restriction sites respectively and cloned in pTZ57R/T vector. Digested fragments were cloned in pRT100 plant expression vector at corresponding sites. After confirmation, the expression cassette of *AdCIPK5* was isolated using *Hin*dIII enzyme and cloned in pCAMBIA2300 vector. The confirmed binary vectors were mobilized into *Agrobacterium* strain EHA105. Tobacco (*Nicotiana tabacum* cv Samsun) was transformed through the standard leaf disc method.

### Molecular analysis of transgenic plants

Transformants were raised on half strength MS media supplemented with 125 mg/l Kanamycin. Plantlets with well-developed root system were shifted to soil cups for acclimatization and later allowed to grow in green house for maturation and seed collection. Putative transgenic plants were screened by PCR for the presence of *AdCIPK5* and *npt*II transgenes using gene-specific primers. The transcript level of *AdCIPK5* was checked in different lines by using semi-quantitative RT-PCR reactions. Mendelian segregation analysis was performed for Kanamycin resistance and sensitive seedlings at seed germination stage.

### Evaluation of fungal resistance in transgenic plants

The fully expanded detached leaves from transgenic and WT control plants were used for the anti-fungal bioassay in T_2_ generation. Fungal resistance was checked against the pathogen, *Phytophthora parasitica* pv *nicotianae*, which causes black shank disease on tobacco. Abrasions were made on the adaxial surface of the leaves and actively growing fungus along with potato dextrose agar block (0.5 cm^2^) was inoculated on abraded areas. The lesions were observed regularly, photographed after 5 days post-inoculation (dpi) and the percentage diseased leaf areas (DLA) were calculated as described earlier^55^. Additionally, cell death was quantified using the Evans blue dye^56^. In brief, equal size of the leaf discs (1 cm^2^) were cut out from WT and transgenic leaves post 5 dpi and submerged into 0.25% (w/v) Evans blue solution for 30 mints. The stained discs were washed with Mili-Q water to remove the unbound dye. Further, the discs were ground in 1% SDS solution and centrifuged at 12000 rpm for 10 min. The supernatant was collected and optical density (OD) was measured at 600 nm of wavelength for cell death measurement.

### Seed germination assay

Mature WT and transgenic seeds were surface sterilized with 4% sodium hypochlorite solution and washed properly with sterile distilled water. From each T_2_ generation transgenic lines, around 150 seeds were taken and transferred to two separate sets of half strength MS media without organics supplemented with 200 mM NaCl and 300 mM Sorbitol separately. Germination was observed regularly and graphs were plotted after 7 d in both treatments. Control plates without any stress agent were also maintained simultaneously.

### Seedling assay

Surface sterilized transgenic seeds were grown on 125 mg/l Kanamycin for 10 d. Simultaneously WT seeds were grown on medium without Kanamycin and maintained at 27 ± 1 °C with a photoperiod of 16 h light and 8 h dark. The seedlings from each of the transgenic plants along with the WT were used in different stress treatments, 200 and 300 mM each of NaCl and sorbitol.

### Leaf disc assay

Leaf discs from the leaves of 7-week-old potted WT and transgenic tobacco plants were used in stress treatments with 100, 200 and 300 mM concentrations of NaCl and 300, 400 and 500 mM concentration of Sorbitol. The treatments were carried out in continuous white light at 27 ± 1 °C until visible differences were observed among the lines.

### Catalase (CAT) and H_2_O_2_ measurement

The 10 d old WT and transgenic seedlings were transferred to media with different concentrations of NaCl and Sorbitol. In the case of NaCl treatment of the transgenics, both CAT and H_2_O_2_ were measured after 6 d, while these measurements were made after 10 d of treatment in the case of Sorbitol. The CAT activity was measured spectrophotometrically by following the oxidation of H_2_O_2_ at 240 nm^57^. The reaction mixture contained 50 mM sodium phosphate buffer (pH 7.0), 20 mM H_2_O_2_ and enzyme extract equivalent to 10 µg protein in a final volume of 1 ml. Δε for H_*2*_O_2_ at 240 nm was 43.6 mM^−1^ cm^−1^.

For H_2_O_2_ measurement, 150 mg seedlings samples (with and without NaCl treatment) were homogenized in 5 ml cold acetone and centrifuged at 1,250 g. Activated carbon was used for the adsorption of chlorophyll contents. The supernatant (200 µl) was added to 1 ml reaction buffer (0.25 mM FeSO_4_, 0.25 mM (NH_4_)_2_SO_4_, 25 mM H_2_SO_4_, 1.25 mM Xylenol orange, and 1 mM Sorbitol) at room temperature for 1 h. The H_2_O_2_ levels were quantified at the 560 nm absorbance as described earlier^58^.

### Quantification of Na^+^ and K^+^ ion

Na^+^ and K^+^ ions were quantified by referring to the methods described by Deng *et al*.^33^. and ion estimation was performed using an inductively coupled Plasma Atomic Emission Spectrometer. The 10 d old WT and transgenic seedlings were transferred to a medium supplemented with 200 mM NaCl for 6 d. A NaCl-free control plate was maintained simultaneously.

### Total chlorophyll and TBARS measurement

Total chlorophyll content and lipid peroxidation were measured by following Arnon^59^ and, Heath and Packer^60^ respectively. In seedling experiments, a 50 mg of sample was used from each line for chlorophyll estimation, while 100 mg tissue was used for both chlorophyll and TBARS analysis in leaf discs experiments.

### ROS detection

By using the epidermal peels from the abaxial surface of fully expanded WT and transgenic leaves, ROS levels were measured using the fluorescence substrate H_2_DCFDA and confocal microscopy (kex-488 nm and kem-530 nm). The experiment was repeated thrice with at least n = 100 stomata. Fluorescence quantification was performed using Image-J 1.42 software by selecting appropriate pigmentation areas.

### Statistical analysis

Statistical analyses were conducted using SIGMASTAT version 11.0. One-way ANOVA (Duncan’s Multiple Range Test) was performed to confirm the variability of results and to determine significant differences between treatment groups. P ≤ 0.05 was considered significantly different with each other and were represented by different letters.

## Supplementary information

Supplementary file.
