# Supplementary material for: A CBL-interacting protein kinase AdCIPK5 confers salt and osmotic stress tolerance in transgenic tobacco
Source: Sci Rep. 2020 Jan 15;10:418. doi: 10.1038/s41598-019-57383-x (PMC6962456; doi:10.1038/s41598-019-57383-x)
Supplement: Supplementary file 1 — Supplementary file. [file 41598_2019_57383_MOESM1_ESM.pdf]

A CBL-interacting protein kinase *AdCIPK5* confers salt and osmotic stress tolerance in transgenic tobacco

Naveen Kumar Singh[1, 2*], Pawan Shukla1 [1, 3], P. B. Kirti[1, 4]

***Corresponding author at:** naveenks11@gmail.com (Naveen Kumar Singh)

[1] Department of Plant Sciences, School of Life Sciences, University of Hyderabad, Hyderabad-500046, India

[2] Agricultural Research Organization-the Volcani Center, 68 HaMaccabim Road P.O.B 15159 Rishon LeZion 7505101, Israel

[3] Central Sericultural Research and Training Institute, Central Silk Board, NH-1A, Gallandar, Pampore - 192 121, J & K, India

4 Agri Biotech Foundation, Rajendranagar, Hyderabad-500030, India

Journal: Scientific Reports

Figure S1. Nucleotide sequence showing 2,031 bp long *AdCIPK5* c-DNA sequence with 5' and 3' UTR sequences in lower case. Start and stop codons are mentioned in bold letters.

agcccaatcccaatcccaatcccaatctcaatatattccccctcactccattaataaattccatcacaacacaaacttcttcaactttcactcaacctaacccaaaaacggaaa
aaccatacccttatttttcccttcttttcttctttctctcttgcacgtacatacacaccggagttccaatctggtgtatcacctatgcaccatgaagatcttcaacccttctgaatcct
ttctccgaaacgttgtgtgtccctcggacactatattcctcaaaggaatgaacctctccaatcttcaccactccacattctccctcctctcttggccatacaattagccttcccaa
ccactaatcttaactaactttctttcttttccttttctctctctggaatcttcctccc**ATG**GATCTGAAAAACGAGATGGAGATCAAGTTGTCG
AACAGCGCTAGGACGGTGATATTCAACAAGTACGAGATGGGACGCGTGTTAGGTCAGGGGAATTTTG
CCAAGGTATACTACAGCAGGAACCTAGCGACGAACGAAAGTGTGGCGATTAAGGTGATAAAGAAGGA
TAAGCTGAGGAAGGAGAGGCTGATAGAGCAGATTAAGAGAGAGGTTTCGGTTATGAGGCTGGTGAGG
CATCCGAACATAGTGGAGCTGAAGGAAGTCATGGCCACGAAGGGGAAGATATTCTTGGTTATGGAGT
ATGTTAAGGGAGGCGAATTGTTCAAGAAAGTCGAGAAAGGGAAGCTCAAGGAAGATGTAGCTAGAAA
GTATTTTCAGCAGTTGATCTCGGCCGTTGATTTCTGCCATAGCCGCGGCGTCACTCACAGAGATCTCAA
GCCGGAGAATCTGCTGCTTGATGAGAATGAAGAGCTTAAGGTCTCCGATTTTGGCCTCTCTGCTTTGCC
GGAACAACGCCGTGCAGATGGTATGCTGATAACACCGTGTGGAACACCTGCATATGTGGCACCAGAA
GTGTTGAAGAAGAAAGGGTATGATGGATCCAAAGCAGATATATGGTCTTGTGGAGTGATTCTTTATGC
TCTGGTTTGTGGCTATCTACCCTTCCAAGGCGAAAACGTGATGAGAATCTACAGAAAAGCCTTCAAGG
CTCAGTATGAATTCCCTGATTGGGTTCCGGAAGGAGCAAAGAACTTGATCTCAAACCTACTTGTAGCT
GATCCTGAAAAGAGGTTCTCAATTGCAGATATCATGAAGGATCCTTGGTTCCAAGTCGGCTTCATGCG
TCCGATTGCATTCTCCTTTAAGGAGTCTGCCATTGGGGACAATGTGGATGACTTCGATGAGGATGATA
GTGATAGTAATAACAATCAAGAGTTTAACAAGGATGATTTGAAGCAGAGTAGTTCTGCTAAGCCTGCG
CGGCCGTTTTACAATGCTTTCGAGATAATTTCCTCGCTGTCGCATGGTTTTGATCTGAGGAGTTTGTTTG
AGACAAGGAAGAGGTCACCTTCAGTGTTTATATCAAAGCTTTCGGCTCAAGCAGTGGTGGGGAAGCTA
GAGGCTATGGCGAAGAAATTGAATTTCAGAGTGGCCGGGAAGAAGAAGAAGGAGTTTGTTGTGAGGA
TGGAAGGGGAGAGGGAAGGGAGGAAAGGGAGGCTGGCCATGACGGTGGAAGTGTTCGAGGTGGCGC
CGGAGGTGGCCGTGGTGGAGTTCTCGAAGTCTTCTGGGGATACCTTGGAGTATATCAAATTCTGTGAG
GAAGAAGTTAGGCCTTCATTGAATGACATTGTTTGGAGTTGGCAGGGAGATGGTAACAAC**TAA**taatacta
gtcatgccattgggattattttgcctatgttgttcttccttttttactgtttgatgcagctatataataatatagtttgatttgcataataagtaaaagaaaaggaagatgctaggaaag
aaataatgctgtattttgattttagttacaggatttcatttcatttgtgtaaaaaatttgagtttagtttctgatgatgtttgaagttaatactgaaaaaaaaaaaaaaa

Figure S2. Alignment of deduced amino acid sequences of AdCIPK5 with closely related CIPKs from other plant species. Adu: *Arachis duranensis*; Gm: *Glycine max*; At: *Arabidopsis thaliana*.

```
GmCIPK5     1 --------------------MDRRSHPTGTV--STRNILFNKYEIGKLLGQGNFAKVYHG
AdCIPK5     1 --------------------MDLKNEMEIKLSNSARTVIFNKYEMGRVLGQGNFAKVYYS
AduCIPK5    1 --------------------MDLKNEMEIKLSNSARTVIFNKYEMGRVLGQGNFAKVYYG
ATCIPK16    1 ----------------------------MEESNRSSTVLFDKYNIGRLLGTGNFAKVYHG
ATCIPK5     1 --------------------ME-----------EERRVLFGKYEMGRLLGKGTFAKVYYG
ATCIPK25    1 MGSKLKLYPLLNHSSVFHPDSRYQSAPTMEEEQQQLRVLFAKYEMGRLLGKGTFGKVYYG

GmCIPK5    39 RNLSTNESVAIKVIKKERL-QKERLVKQIKREVSVMRLVRHPHIVELKEVMANKAKIFMV
AdCIPK5    41 RNLATNESVAIKVIKKDKL-RKERLIEQIKREVSVMRLVRHPNIVELKEVMATKGKIFLV
AduCIPK5   41 RNLATNESVAIKVIKKDKL-KKERLIEQIKREVSVMRLVRHPNIVELKEVMATKGKIFLV
ATCIPK16   33 TEISTGDDVAIKVIKKDHVFKRRGMMEQIEREIAVMRLLRHPNVVELREVMATKKKIFFV
ATCIPK5    30 KEIIGGECVAIKVINKDQVMKRPGMMEQIKREISIMKLVRHPNIVELKEVMATKTKIFFV
ATCIPK25   61 KEITTGESVAIKIINKDQV-KREGMMEQIKREISIMRLVRHPNIVELKEVMATKTKIFFI

GmCIPK5    98 VEYVKGGELFAKVAK-GKMKEDVARKYFQQLISAVDFCHSRGVTHRDLKPENLLLDENED
AdCIPK5   100 MEYVKGGELFKKVEK-GKLKEDVARKYFQQLISAVDFCHSRGVTHRDLKPENLLLDENEE
AduCIPK5  100 MEYVKGGELFKKVEK-GKLKEDVARKYFQQLISAVDFCHSRGVTHRDLKPENLLLDENEE
ATCIPK16   93 MEYVNGGELFEMIDRDGKLPEDLARKYFQQLISAVDFCHSRGVFHRDIKPENLLLDGEGD
ATCIPK5    90 MEFVKGGELFCKISK-GKLHEDAARRYFQQLISAVDYCHSRGVSHRDLKPENLLLDENGD
ATCIPK25  120 MEYVKGGELFSKIVK-GKLKEDSARKYFQQLISAVDFCHSRGVSHRDLKPENLLVDENGD

                       Activation loop
GmCIPK5   157 LKVSDFGLSAL--PD-----QRRSDGMLLTPCGTPAYVAPEVLKKKGYDGSKADIWSCGV
AdCIPK5   159 LKVSDFGLSAL--PE-----QRRADGMLITPCGTPAYVAPEVLKKKGYDGSKADIWSCGV
AduCIPK5  159 LKVSDFGLSAL--PE-----QRRADGMLITPCGTPAYVAPEVLKKKGYDGSKADIWSCGV
ATCIPK16  153 LKVTDFGLSALMMPEGLGGRRGSSDDLLHTRCGTPAYVAPEVLRNKGYDGAMADIWSCGI
ATCIPK5   149 LKISDFGLSAL--PE-----QILQDGLLHTQCGTPAYVAPEVLKKKGYDGAKADIWSCGV
ATCIPK25  179 LKVSDFGLSAL--PE-----QILQDGLLHTQCGTPAYVAPEVLRKKGYDGAKGDIWSCGI
                       *                    *       *

GmCIPK5   210 ILFALLSGYLPFQGENVMRIYSKSFKADYAFPEWISPAAKNLISNLLVVDPQKRYSIPDI
AdCIPK5   212 ILYALVCGYLPFQGENVMRIYRKAFKAQYEFPDWVPEGAKNLISNLLVADPEKRFSIADI
AduCIPK5  212 ILYALVCGYLPFQGENVMRIYRKAFKAQYEFPDWVPEGAKNLISNLLVADPEKRFSIADI
ATCIPK16  213 VLYALLAGFLPFIDENVMTLYTKIFKAECEFPPWFSLESKELLSRLLVPDPEQRISMSEI
ATCIPK5   202 VLYVLLAGCLPFQDENLMNMYRKIFRADFEFPPWFSPEARRLISKLLVVDPDRRISIPAI
ATCIPK25  232 ILYVLLAGFLPFQDENLMKMYRKIFKSEFEYPPWFSPESKRLISKLLVVDPNKRISIPAI

GmCIPK5   270 MKDPWFQIGFLRPIAFSMKESALEDNI-DFSGDDVPSPGSDDGNSSSNNED-GELTGAKP
AdCIPK5   272 MKDPWFQVGFMRPIAFSFKESAIGDNVDDFDEDD----SDSNNNQEFNKDDLKQSSSAKP
AduCIPK5  272 MKDPWFQVGFMRPIAFSFKESAIGDNVDDFDEDD----GDSNNNQEFNKDDLKQSSSAKP
ATCIPK16  273 KMIPWFRKNFTPSVAFSIDETIP---------------SPPEPPTKKKKKDLNEKEDDGA
ATCIPK5   262 MRTPWLRKNFTPPLAFKIDEPIC---------------SQSSKNNEEEEEDGDCENQTEP
ATCIPK25  292 MRTPWFRKNINSPIEFKIDELEI----------------QNVEDETPTTTATTATTTTTP

                   NAF motif
GmCIPK5   328 ARP-SYNAFEIISSLSHGFDLRNLFETRKRSPSMFISKFSASAVMAKLEGVAKKLNFRVT
AdCIPK5   328 ARP-FYNAFEIISSLSHGFDLRSLFETRKRSPSVFISKLSAQAVVGKLEAMAKKLNFRVA
AduCIPK5  328 ARP-FYNAFEIISSLSHGFDLRSLFETRKRSPSVFISKLSAQAVVGKLEAMAKKLNFRVA
ATCIPK16  318 S-PRSFNAFQFITSMSSGFDLSNLFEIKRKPKRMFTSKFPAKSVKERLETAAREMDMRV-
ATCIPK5   307 ISPKFFNAFEFISSMSSGFDLSSLFESKRKVQSVFTSRSSATEVMEKIETVTKEMNMKV-
ATCIPK25  336 VSPKFFNAFEFISSMSSGFDLSSLFESKRKLRSMFTSRWSASEIMGKLEGIGKEMNMKV-

GmCIPK5   387 GK--KEFMVRMQGATEGRKGKLAMTVEVFEVAPEVAVVEFSKSAGDTLEYLNFCEDQVRP
AdCIPK5   387 GKKKKEFVVRMEGEREGRKGRLAMTVEVFEVAPEVAVVEFSKSSGDTLEYIKFCEEEVRP
AduCIPK5  387 GKKKKEFVVRMEGEREGRKGRLAMTVEVFEVAPEVAVVEFSKSSGDTLEYIKFCEEEVRP
ATCIPK16  376 -KHVKDCKMKLQRRTEGRKGRLSVTAEVFEVAPEVSVVEFCKTSGDTLEYYLFCEDDVRP
ATCIPK5   366 -KRTKDFKVKMEGKTEGRKGRLSMTAEVFEVAPEISVVEFCKSAGDTLEYDRLYEEEVRP
ATCIPK25  395 -KRTKDFKVKLFGKTEGRKGQIAVTAEVFEVAPEVAVVELCKSAGDTLEYNRLYEEHVRP

GmCIPK5   445 SLRDIVWSWQGDANCHQ------------------
AdCIPK5   447 SLNDIVWSWQGDGNN--------------------
AduCIPK5  447 SLNDIVWSWQGDGNN--------------------
ATCIPK16  435 ALKDIVWSWQGDDDEDDVTTNDNVDTNDNKINNVS
ATCIPK5   425 ALNDIVWSWHGDNNN--------------TSSEDC
ATCIPK25  454 ALEEIVWSWHGDNHNNNIVKSNGNYVSDENSGSDC
```

Figure S3. Phylogenetic tree of AdCIPK5 and related protein kinases: AduCIPK5 of *Arachis duranensis*; GmCIPK5 of *Glycine max*; AtCIPK 1–26 of *Arabidopsis thaliana.*

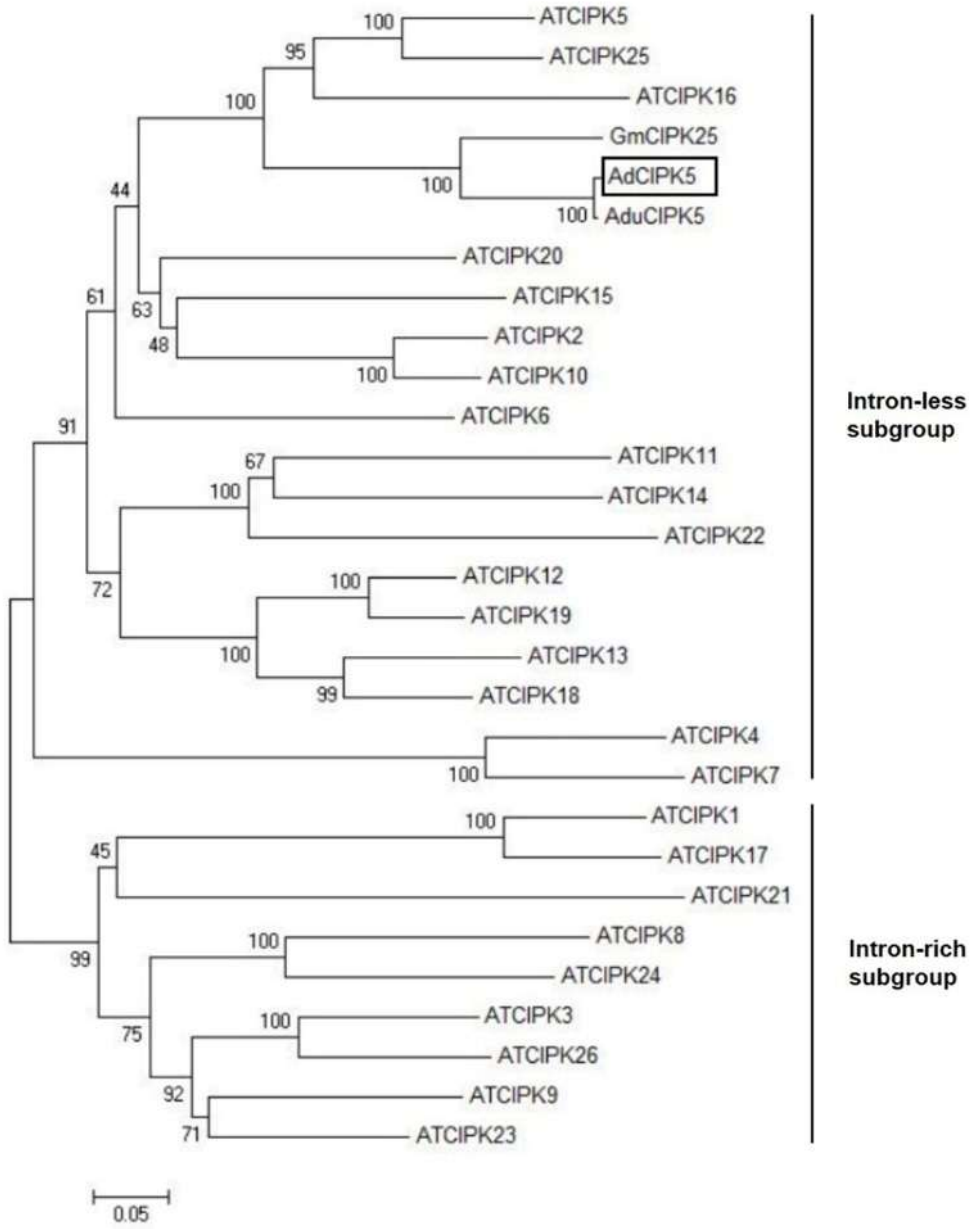

Figure S4. Gel pictures showing 739 bp amplified PCR product of *nptII* (A) and 1386 bp of *AdCIPK*5 ORF (B) from genomic DNA of nine different putative T_0 transgenic lines and WT plants. Letter M represents λ *EcoR*I/*Hind*III DNA ladder.

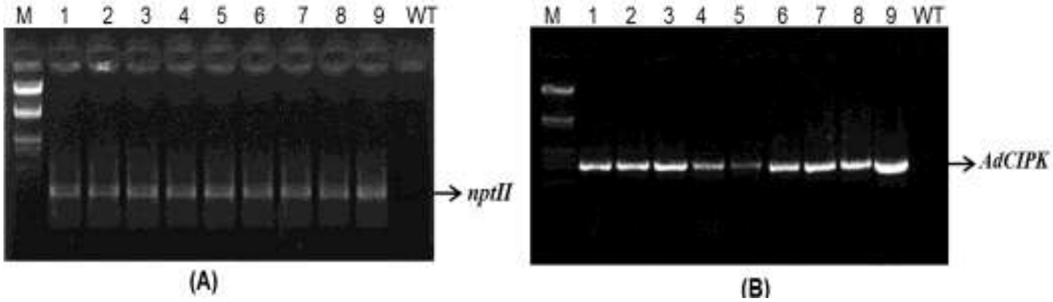

Figure S5. Semi-quantitative RT-PCR analysis of *AdCIPK5* gene in putative T_0 **(A)** and T_2 **(B)** transgenic plants. Lane 1 and 6 represent high expression lines and lane 2 represent the low expression line. *Actin* served as internal control to demonstrate equal loading

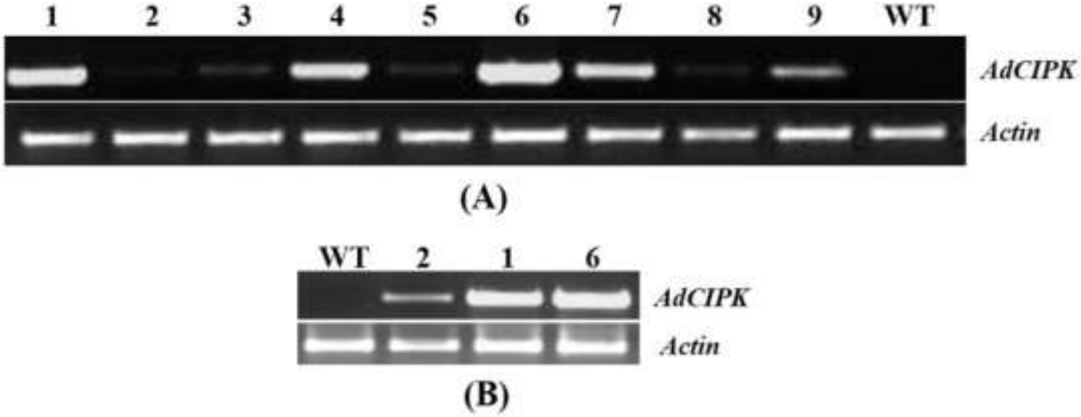

Figure S6. Resistance to fungal infection was checked in mature leaves of WT and T_2 transgenic plants against *Phytophthora parasitica* var. nicotianae after 5 d of treatment **(a)**. No differences were observed between wild type and transgenic lines in terms of damaged leaf area and tissue cell death **(b and c)**. Data represent means ±SE of three replicates and same letters indicate no significant difference among the lines.

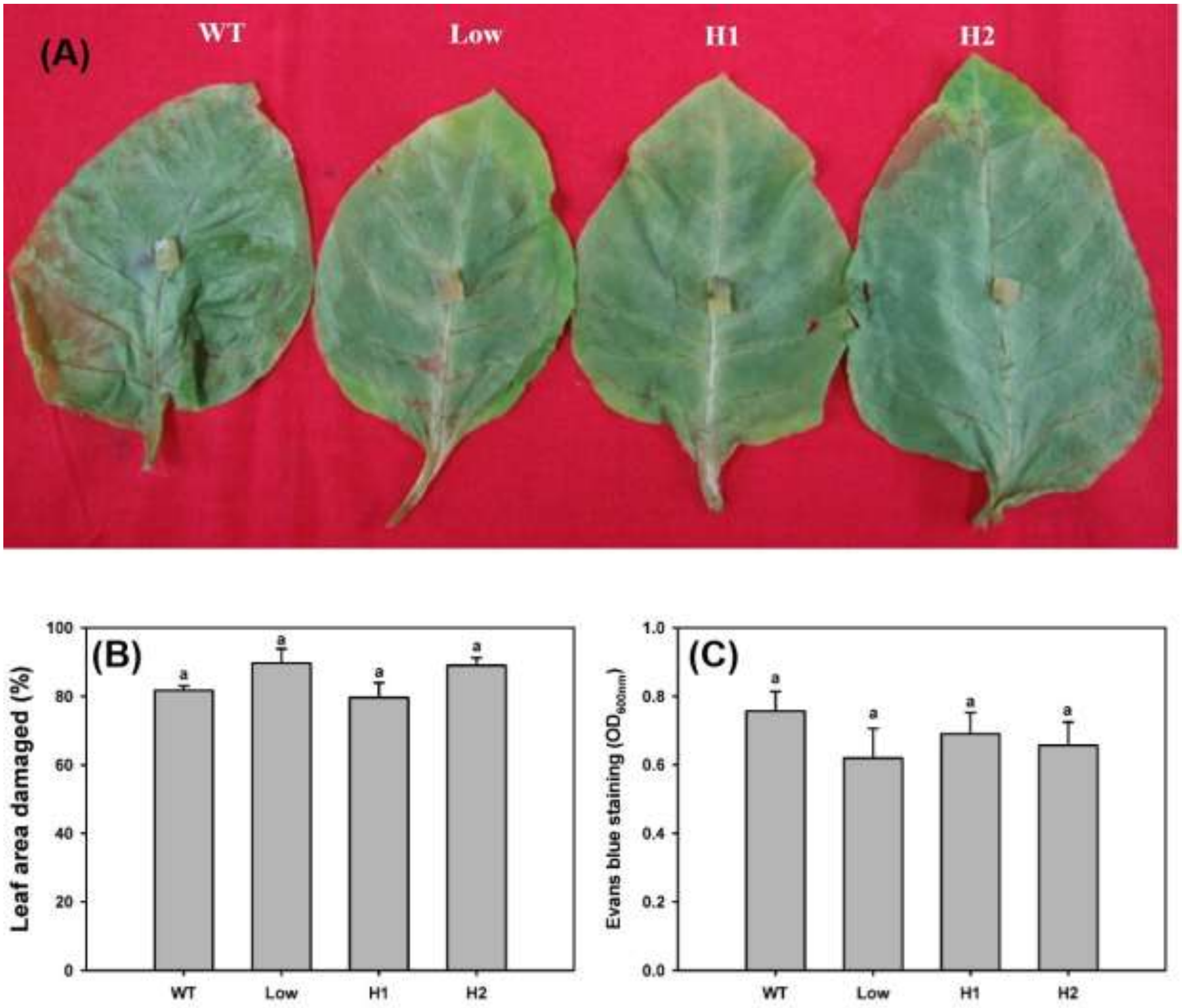

Table S1. Sequences of the oligonucleotides used for RACE, cloning and PCR analysis (see text for details).

**Name of the primers**	**Primer sequences (5′-3′)**
SMARTer II A oligo	AAG CAG TGG TAT CAA CGC AGA GTA CGC GGG
5′ CDS	5′-$(T)_{25}$ V N-3′ (N=A, C, G or T; V= A, G or C)
3′ CDS	AAGCAGTGGTATCAACGCAGAGTAC$(T)_{30}$ V N (N=A, C, G or T; V=A, G or C)
Universal Primer A Mix	Long 5′-CTAATACGACTCACTATAGGGCAAGCAGTGGTATCAACGCAGAGT-3′ Short 5'-CTAATACGACTCACTATAGGGC-3'
Nested Primer	AAGCAGTGGTATCAACGCAGAGT
5′GSP-1	AGAACTACTCTGCTTCAA
5′GSP-2	CATCCTCATCGAAGTCATCCA
5′GSP-3	AAAGGAGAATGCAATCGGACG
3′ GSP1	TGGATGACTTCGATGAGGATG
ORF-F1	GCCATGGATCTGAAAAACGAGATGGA
ORF-R1	CCACTAGTGTTGTTACCATCTCCCT
ORF-F2	CCGGGCCCATGGATCTGAAAAACGAGATGGA
ORF-R2	CCGGTACCTTAGTTGTTACCATCTCCCTGC

Table S2. Sequences of the oligonucleotides used in qRT-PCR (see text for details). Nt: *Nicotiana tabacum*; Ad: *Arachis diogoi*.

**Oligo name**	**Forward (5′-3′)**	**Reverse (5′-3′)**
NtCAT	GGCCGCTACAACTCTCTCTTT	ACAGGACCTCTTGCACCAAC
NtERD10C	AAAGCCAACTCATGCCCAAG	AGAGCTGCTACTTGATCGATGG
NtERD10D	GCACGAGGGAAGAAGAGAAGG	TGGAGGCGCCACTTCCTC
NtNCED1	TGTCTGAAATGATCCGGGGC	AGTTTCCGGCTCTTCCCAAG
NtSUS1	CACGGATATTTCGCCCAGGA	GCAGCAGCCGAGTAGCAATA
NtSOS1	CAAATGTTATCCCCCGAAAGC	CGGAGAACCTGAGGAAATGTGA
NtUbq	GAGTCAACCCGTCACCTTGT	ACATCTTTGAGACCTCAGTAGACA
Adadh3	GACGCTTGGCGAGATCAACA	AACCGGACAACCACCACATG
Adcipk	CCATGACGGTGGAAGTGTTC	TACCATCTCCCTGCCAACTC
